# Integrated analysis of miRNA-mRNA expression of newly emerging swine H3N2 influenza virus cross-species infection with tree shrews

**DOI:** 10.1186/s12985-023-02260-3

**Published:** 2024-01-04

**Authors:** Qihui Wang, Zihe Liu, Xia Zeng, Yu Zheng, Li Lan, Xinhang Wang, Zhenping Lai, Xiaoqiong Hou, Lingxi Gao, Liang Liang, Shen Tang, Zengfeng Zhang, Jing Leng, Xiaohui Fan

**Affiliations:** 1 Department of Immunology, Guangxi Medical University, Nanning, 530021 China; 2 Department of Microbiology, Guangxi Medical University, Nanning, 530021 China; 3 Key Laboratory of Basic Research on Regional Diseases (Guangxi Medical University), Education Department of Guangxi Zhuang Autonomous Region, Nanning, 530021 China; 4 Guangxi Key Laboratory of Translational Medicine for Treating High-Incidence Infectious Diseases with Integrative Medicine, Guangxi University of Chinese Medicine, Nanning, 530200 China; 5 Key Laboratory of Characteristic Experimental Animal Models of Guangxi, Guangxi University of Chinese Medicine, Nanning, 530200 China

**Keywords:** Swine influenza virus, Tree shrew, Cross-species infection, miRNA-mRNA, Bioinformatics, Turbinate tissue

## Abstract

**Background:**

Cross-species transmission of zoonotic IAVs to humans is potentially widespread and lethal, posing a great threat to human health, and their cross-species transmission mechanism has attracted much attention. miRNAs have been shown to be involved in the regulation of IAVs infection and immunity, however, few studies have focused on the molecular mechanisms underlying miRNAs and mRNAs expression after IAVs cross-species infection.

**Methods:**

We used tree shrews, a close relative of primates, as a model and used RNA-Seq and bioinformatics tools to analyze the expression profiles of DEMs and DEGs in the nasal turbinate tissue at different time points after the newly emerged swine influenza A virus SW2783 cross-species infection with tree shrews, and miRNA-mRNA interaction maps were constructed and verified by RT-qPCR, miRNA transfection and luciferase reporter assay.

**Results:**

14 DEMs were screened based on functional analysis and interaction map, miR-760-3p, miR-449b-2, miR-30e-3p, and miR-429 were involved in the signal transduction process of replication and proliferation after infection, miR-324-3p, miR-1301-1, miR-103-1, miR-134-5p, miR-29a, miR-31, miR-16b, miR-34a, and miR-125b participate in negative feedback regulation of genes related to the immune function of the body to activate the antiviral immune response, and miR-106b-3p may be related to the cross-species infection potential of SW2783, and the expression level of these miRNAs varies in different days after infection.

**Conclusions:**

The miRNA regulatory networks were constructed and 14 DEMs were identified, some of them can affect the replication and proliferation of viruses by regulating signal transduction, while others can play an antiviral role by regulating the immune response. It indicates that abnormal expression of miRNAs plays a crucial role in the regulation of cross-species IAVs infection, which lays a solid foundation for further exploration of the molecular regulatory mechanism of miRNAs in IAVs cross-species infection and anti-influenza virus targets.

## Introduction

Influenza A viruses (IAVs) are single and negatively stranded with rapid and frequent mutation and recombination rates, resulting in the emergence of new subtypes with unpredictable pathogenicity and transmissibility [1]. In general, IAVs have the characteristics of species-specific, however, some viruses of certain species can spread to humans across species and genera. IAVs cause approximately 500,000 deaths worldwide every year, in addition, cross-species influenza viruses from poultry and pigs have caused many deaths, posing a serious threat to human health [2–5]. Pigs are considered as intermediate hosts and “mixing vessels” for influenza viruses, and the 2009 H1N1 pandemic virus (pdm/09) was generated by multiple recombination of swine, human, and avian influenza viruses, infecting not only humans but also pigs, and became part of the swine influenza viruses (SIVs) gene pool after further recombination with other SIVs gene fragments [6, 7]. The SIV strain SW2783 we selected not only has a recombinant pdm/09-M gene, but also is highly related to the gene sequence of the H3N2 triple recombinant virus (TRV) strain (Vietnam/302/11) isolated from a Vietnamese girl with influenza [8]. In addition, previous research showed that SW2783 had the ability to infect human lung tissue in vitro and replicate and multiply in tree shrews to cause lesions without adaptation, spread between tree shrews and from tree shrews to guinea pigs, and stimulate better innate and adaptive immune responses [9]. It suggests that SW2783 has the potential to infect humans. Due to the widespread presence of pigs as natural hosts, SW2783 may have higher transmissibility and fatality rates if it is further recombined with other IAVs in pigs to create new subtypes, putting the risk of re-outbreak of this subtype of IAVs at any time. In addition, owing to the frequent occurrence of antigen drift and transfer as well as the continuous emergence of drug-resistant strains, it is particularly important to quickly explore the pathogenesis and develop more effective methods for prevention and treatment.

The establishment of experimental animal models is extremely important for studying viral transmission, infection, pathogenesis, and immune responses of the host [10]. A variety of animal models have been developed for IAVs, including mice, guinea pigs, ferrets, and non-human primates (such as macaques), each with their own advantages and disadvantages [11]. The animal model selected in this study is tree shrew (*Tupaia belangeri*), which has genetically closer to humans than rodents in terms of genetic relationships and phylogenetic development, and is often considered a lower primate. Sequencing of tree shrews genome has also confirmed that it has a high affinity with primates [12, 13]. It has been pointed out that tree shrews are very similar to humans in physiology, biochemistry and immune regulation, and lack of the important antiviral gene RIG-I, making them a suitable animal model for studying viruses infection. Study results showed that the distribution of IAVs sialic acid receptors of respiratory in tree shrews is analogous to that of humans and ferrets, and they would present various pathological changes and clinical symptoms after being infected with IAVs [14, 15]. In addition, they are small in size and reproduce under artificial conditions similar to those of rats, with a short cycle and lower cost than ferrets and non-human primates (such as macaques). Therefore, they have received extensive attention in the establishment of animal models for research on human diseases and virus infection [16, 17]. So far, several studies both domestically and internationally have used tree shrews as animal models of different types of influenza viruses infection, suggesting that they are more susceptible to infection and can show clinical symptoms similar to humans, which can provide a scientific animal model for studying the infection mechanism and prevention and treatment of influenza viruses [9, 18].

MicroRNAs (miRNAs) are small endogenous non-coding RNA of 21-22nt, which are known to be post-transcriptional regulators of animals, plants and some viruses. Studies have shown that a single miRNA can exert extensive and significant influences on physiological and pathological processes of the body, and alterations in its function can lead to biological dysfunction and diseases [19]. Researches on dysregulated miRNAs plays a key role in the progression of various diseases, such as aging, infectious diseases, autoimmune diseases, cancer and viral infections such as HPV and HIV, which has opened new avenues for research on anti-influenza virus targets [20, 21]. In recent years, many studies have reported that the miRNAs regulatory networks constructed by the hosts and viruses are indispensable in the process of viruses infection and transmission, and the miRNAs expression profiles of the host changes after IAVs infection, which plays a role in inhibiting the replication and transcription of the influenza viruses through specific combinations with virus genes and regulating the antiviral immune response [22, 23]. Therefore, it is of great significance to begin with miRNAs that play a special regulatory role in the pathogenesis, prevention, and treatment of influenza viruses infection.

The previous research have observed the histopathology of tree shrews infected with SW2783, and found that it mainly caused upper respiratory turbinate tissue infection [9]. Therefore, in this study, we conducted global miRNA and mRNA expression profiles analysis on turbinate tissue cells of tree shrews infected with the newly emerged swine influenza A virus SW2783 and explored the similarities and differences of miRNAs molecular regulation mechanisms at three different time points (3, 5 and 7 days post infection (dpi)). We screened miRNAs involved in influenza viruses replication and immune response regulation and used real-time quantitative PCR (RT-qPCR) to determine whether the expression of miRNAs and mRNAs was consistent with the sequencing results. It not only provides differentially expressed miRNAs (DEMs) and mRNAs (DEGs) after infection but also provides detailed miRNAs regulatory networks, targeted genes, Gene Ontology (GO), and Kyoto Encylopaedia of Genes and Genomes (KEGG) enrichment analyses, which provide new insights into the anti-IAVs mechanism. The selected miRNAs are the prelude to the biological progress of tree shrews infected with IAVs, and the use of tree shrews as model animals for the study of human influenza viruses infection provides a basis for further study of the role of miRNAs in IAVs infection and can provide a reference for the study of broad-spectrum antiviral targets to guide the prevention and control of influenza pandemics.

## Materials and methods

### Virus strains, animals and cells

The virus used in this study was a newly emerged H3N2 subtype of swine influenza virus A/swine/Guangxi/NS2783/2010 (H3N2) (abbreviation: SW2783), with its genome integrated with the gene fragment of the 2009 new H1N1 pandemic virus (pdm/09), which was preserved in our laboratory. Healthy adult tree shrews of the West Yunnan Asian species were purchased from the Department of Experimental Zoology of Kunming Medical University. Animal experiments were carried out in strict accordance with the animal protection and use system and rules of the Experimental Animal Ethics Committee of Guangxi Medical University. Approval Code: 202008013, Approval Date: 2020.08.13. 293T and MDCK cells lines were purchased from Shanghai Institutes for Biological Sciences (Shanghai, China), grown in DMEM(Gibco), supplemented with 1% Penicillin-Streptomycin Liquid (100×) (Solarbio) and 10% heat-inactivated FBS (Gibco).

### Virus infected tree shrews and total RNA extraction

Twelve tree shrews were isolated for one week and then took nasopharyngeal swabs and inoculate them with chicken embryos and MDCK for hemagglutination (HA) test to detect the presence of IAVs infection. Blood samples collected from the tail vein were taken for centrifugation and sera were collected for the haemagglutination inhibition (HI) test to ensure that there were no IAVs antibodies in the test animals. Tree shrews were randomly divided into two groups: nine in the SW2783 group (three in each of the 3, 5 and 7 dpi groups) and three in the negative control (NC) group; 3% pentobarbital sodium was intraperitoneally injected according to the weight ratio (0.2 mL/100 g) for anaesthesia. The virus solution, diluted with PBS to 10^5^ TCID_50_/mL was used for infection experiments through the nose, eyes, and pharyngeal tonsils. Each tree shrew was inoculated with 250 μL of virus diluent, 200 μL into the nasal cavity (100 μL/nasal cavity), 20 μL into the eye conjunctiva (10 μL/eye), 30 μL into the tonsils (15 μL/tonsil). The NC group was given 250 μL PBS and the dosage was the same as that in the experimental group. The turbinate tissues of tree shrews were collected aseptically and divided into two parts, which were quickly frozen in liquid nitrogen for several hours and then stored at − 80 °C. One part was used for RNA extraction and determination of relevant immune molecules, and the other part was analysed by high-throughput sequencing (RNA-seq).

Total RNA was extracted by TRIzol. Chloroform was added to extract the upper aqueous phase after homogenising the tissue samples, and RNA was precipitated with isopropyl alcohol and 75% ethanol. After drying, 50 μL RNase-free water was added to dissolve the RNA in a water bath, and the RNA was quantified using NanoDrop and stored at − 80 °C.

### miRNA sequencing and expression profiling analysis

miRNA sequencing was performed using BGISEQ-500 by BGI Wuhan, and SOAPnuke which was developed independently by BGI, was used for filtering. Next, AASRA was used to compare the clean tags with the reference genome sequence of the species to determine the location of the fragments originating from the genome. Finally, the miRNAs were quantified and the output result was an expression matrix with a count value. Differential analysis of miRNAs was performed using the DESeq2 package in R, with screening conditions Fold Change ≥ 2 and Adjust *P*-value ≤ 0.05. The DEMs were displayed in a volcano map and clustered in a heat map. The target genes of the DEMs were predicted using RNAhybrid and miRanda software, and GO and KEGG pathway enrichment of them were performed using the clusterProfiler package based on the hypergeometric distribution in R.

### mRNA sequencing and expression profiling analysis

mRNA sequencing was also performed by BGI. After sequencing and filtering, HISAT2 was used to compare clean reads with the reference genome sequence of the species to determine the location of the genome from which the fragments originated. Gene expression was calculated using the expected fragments per kilobase of transcript per million fragments sequenced (FPKM) method. The method of differential analysis of genes was the same as that of miRNA. Cluster analysis, enrichment analysis of GO and KEGG, and KEGG orthology (KO) analyses were performed on DEGs.

### Cross-linking analysis of miRNA and mRNA

Venn Diagram is a graph that uses a closed curve to visually represent a set and its relationship. It is often used to show the mathematical or logical connection between different groups of things (sets), and the data from different groups in the experiment are intersected, and the research direction is selected through the information of different data intersection [24]. First, the Venn Diagram package was used to analyze DEMs and DEGs in the 3, 5 and 7 dpi groups to verify whether miRNA regulatory gene expression was timeliness after IAVs infection. The intersection of the target genes predicted by DEMs and DEGs was determined using R. Next, the gene database of tree shrews was downloaded using the AnnotationHub package in R to screen for miRNA-mRNA interaction pairs related to viral replication and immune regulation after SW2783 infection. Finally, the miRNA-mRNA regulatory network was mapped using Cytoscape.

### Detection of DEMs and DEGs by RT-qRCR

To validate the high-throughput sequencing results, three miRNAs and three mRNAs were selected for the RT-qPCR analysis. Total RNA was extracted from the NC group and the three experimental groups with different infection times and then reverse transcribed to synthesise cDNA. RT-qPCR primers were designed by NCBI primer blast and synthesised by Sangon Bioengineering (Shanghai) Co., Ltd. RT-qPCR was performed using ABI 7500 fluorescence quantitative PCR instrument and the reaction mixture was incubated at 95 °C for 30 s in 384-well optical plates (Roche, Swiss), followed by 40 cycles of incubation at 95 °C for 5 s, 60 °C for 10 s, and 72 °C for 30 s. Each sample was tested in triplicate, and the relative expression was calculated using 2^−△△Ct^ [25].

### Cell culture, transfection and relationship validation

In order to confirm the targeted regulatory relationship between the screened miRNAs and mRNAs, the simple random sampling method was used to select two miRNAs and seven target genes from the interaction networks with random numbers generated in Microsoft Excel. Specific miRNA mimics and mimics NC were synthesized by GenePharma Shanghai, and 293 T cells were transfected with Lipofectamine™ 3,000 (Thermo Fisher Scientific). Then RT-qPCR was performed to detect miRNA and mRNA expression according to the same method mentioned above, and U6 and β-actin were selected as internal parameters of miRNA and mRNA. The used primers were listed in Table 1.

**Table 1:** Primers used for RT-qPCR

RNA	Forward primer	Reverse primer
miR-324-3p	ATAGATTCCCACTGCCCCAGGT	ATCCAGTGCAGGGTCCGAGG
miR-449b	CCTACTACCAGCCACAACTACCC	ATCCAGTGCAGGGTCCGAGG
miR-30a-5p	GGCGGTGTAAACATCCTCG	ATCCAGTGCAGGGTCCGAGG
miR-181e	AACATTCATTATCAGACTG	ATCCAGTGCAGGGTCCGAGG
MEF2B	ATGGGGAGGAAAAAAATTCA	CTACCGGGGCCAGCCATCAG
MUC5AC	ACAGGACTAGCAGCAGTA	CCATAGCAGTAGACAAG
S100A12	ATGACTAAGCTGGAAGACCATC	CTACTCTTGTGGATGTTGTC
CD6	CAGCCCTCTCAGGTCATCCA	CTCCCGTTTGTCAGACGGAC
LTF	ATGGTGGTTTCATATACGAGGCA	CTTTCGGTCCCGTAGACTTCC
TNFAIP2	GGCCAATGTGAGGGAGTTGAT	CCCGCTTTATCTGTGAGCCC
SELE	CAGCAAAGGTACACACACCTG	CAGACCCACACATTGTTGACTT
RND2	ACACTGCGAGCTTTGAGATCG	AGAGGCCGGACATTATCATAGT
RRM2	GTGGAGCGATTTAGCCAAGAA	CACAAGGCATCGTTTCAATGG
CRMP1	CAACGGGCGGATGGTTATTC	CTCGAAAGAGGTCAGTAGGCT
KIF1A	GTCCGCCCCTTCAATTCCC	GAGGTGTGCGACCAGTAGG
PSMB9	GCACCAACCGGGGACTTAC	CACTCGGGAATCAGAACCCAT
CCL20	TGCTGTACCAAGAGTTTGCTC	CGCACACAGACAACTTTTTCTTT
IFIH1	TCGAATGGGTATTCCACAGACG	GTGGCGACTGTCCTCTGAA
KLK10	CAAGGCGAACGGATGAGCA	GAGCACAGCGGTAGGGAAG
IFIT5	GGCCAAAATAAAGACGCCCTT	GACCAGGCTTCGTACTTCTTC
STMN1	GCTCGGACTGAGCAGGACT	CAGAGATCTGTGCTGGGTGG
U6	CCCACTGCCCCAGGTGC	AGTGCAGGGTCCGAGGTATT
β-actin	TCCTCTCCCAAGTCCACACAGG	GGGCACGAAGGCTCATCATTC

### Luciferase reporter assay

LTF wild-type sequences containing miR-324-3p binding site and mutant sequence without miR-324-3p binding site were cloned into GP-miRGLO vectors to construct wild-type reporter vectors (WT) and mutant reporter vectors (MUT), which were completed by GenePharma Shanghai. Then, miR-324-3p mimics, miR-NC and GP-miRGLO were co-transfected into 293 T cells by Lipofectamine™ 3000. 48 h later, the cells were split on ice for 15 min and then centrifuged. Dual-Glo Reporter Assay System (Promega) was used to analyze luciferase activity based on the manufacturer's instructions. The activity of firefly luciferase was normalized to that of renilla luciferase.

### Statistical analysis

Data were expressed as mean ± standard deviation (SD) from at least four independent experiments. SPSS 21.0 software was used for statistical analyses. One-way ANOVA analysis for multiple groups, and the difference between the two groups was determined to be statistically significant at a value of *P* < 0.05.

## Results

### Expression spectrum analysis

In order to explore the changes of gene expression in viral infection and immune response regulation in tree shrews infected with the newly emerging swine influenza A virus SW2783, all miRNAs and mRNAs expressed in turbinate tissue cells in 3, 5 and 7 dpi groups were compared with those in the NC group. DEMs and DEGs were selected under a Fold Change ≥ 2 and Adjust *P* value ≤ 0.05.

**Fig. 1:**
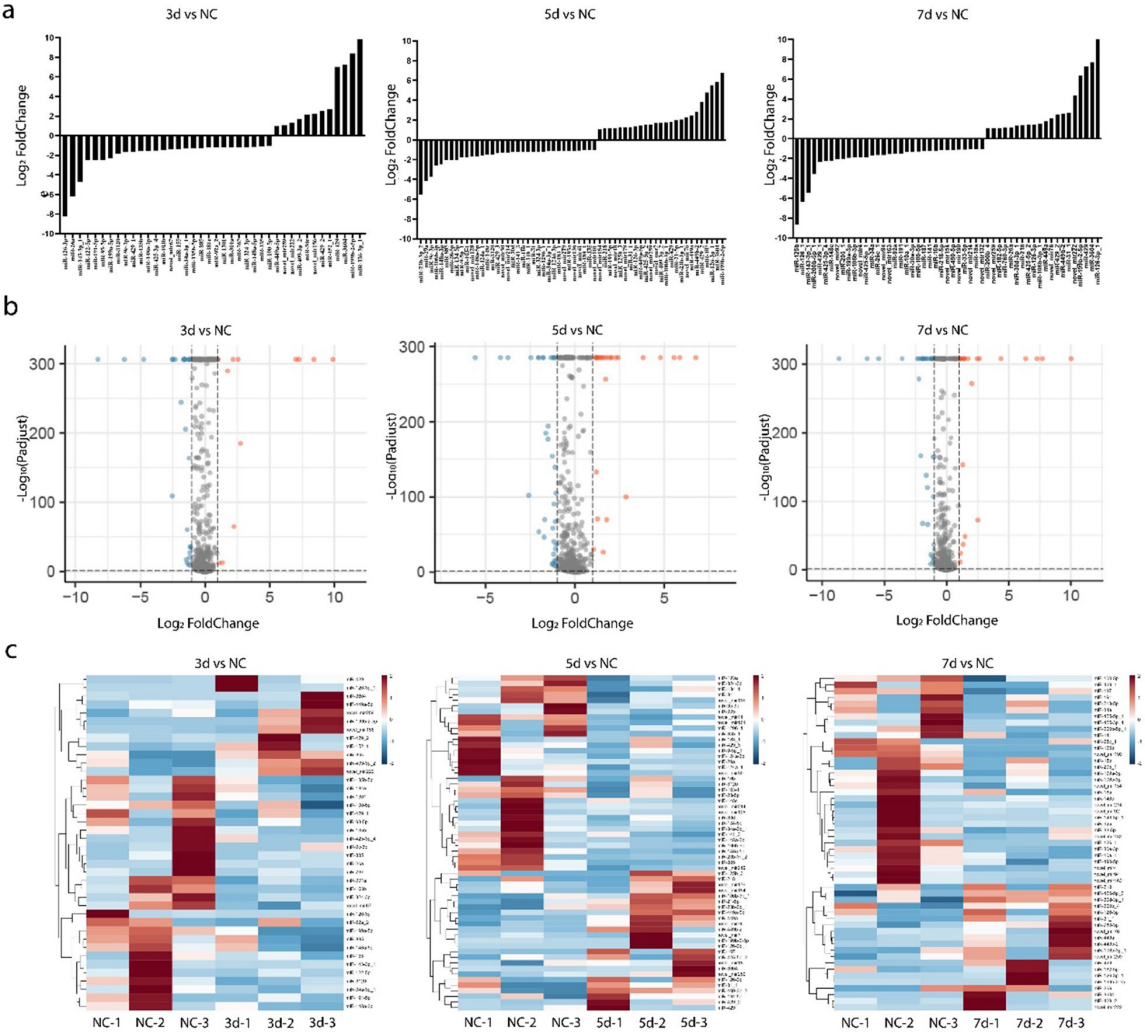
miRNAs expression profiles of SW2783 infected and NC groups. **a** Expression of DEMs. **b** Volcano map of DEMs, gray is the gene with non-significant difference, red and blue are the genes with significant difference (red represents up-regulated, blue represents down-regulated); the X-axis direction is the display of log_2_ Fold Change, and the Y-axis direction is − log_10_ (*P* adjust). **c** Hierarchical clustering Heatmap of DEMs, red represents upregulated, and blue represents downregulated. DEMs, differentially expressed miRNAs; DEGs, differentially expressed genes; 3d, 3 days post infection; 5d, 5 days post infection; 7d, 7 days post infection; NC, negative control

The expression of miRNAs in the infected groups were compared with that in the NC group, and a total of 40, 60 and 53 miRNAs were differentially expressed in the 3 dpi (12 upregulated and 28 down regulated), 5 dpi (25 upregulated and 35 down regulated), and 7 dpi (21 up-regulated and 32 down-regulated) groups, respectively, as shown in Table 2. The expressions of DEMs are plotted as bars in Fig. 1a. More down-regulated miRNAs were detected in the three experimental groups, indicating that the changes in miRNA expression were mainly down-regulated after IAVs infection. A volcano diagram was used to display the expression of the miRNAs that met the screening criteria (Fig. 1b). Hierarchical cluster analysis was performed on the DEMs. The expression trend of the same miRNA at different time points of infection in different tree shrews was basically the same, but the expression levels were different, as shown in the heat map in Fig. 1c.

**Table 2:** Number of DEMs and DEGs

Compare group	Up regulated		Down regulated	
	miRNA	mRNA	miRNA	mRNA
3 dpi vs NC	12	138	28	234
5 dpi vs NC	25	568	35	232
7 dpi vs NC	21	251	32	147

**Fig. 2:**
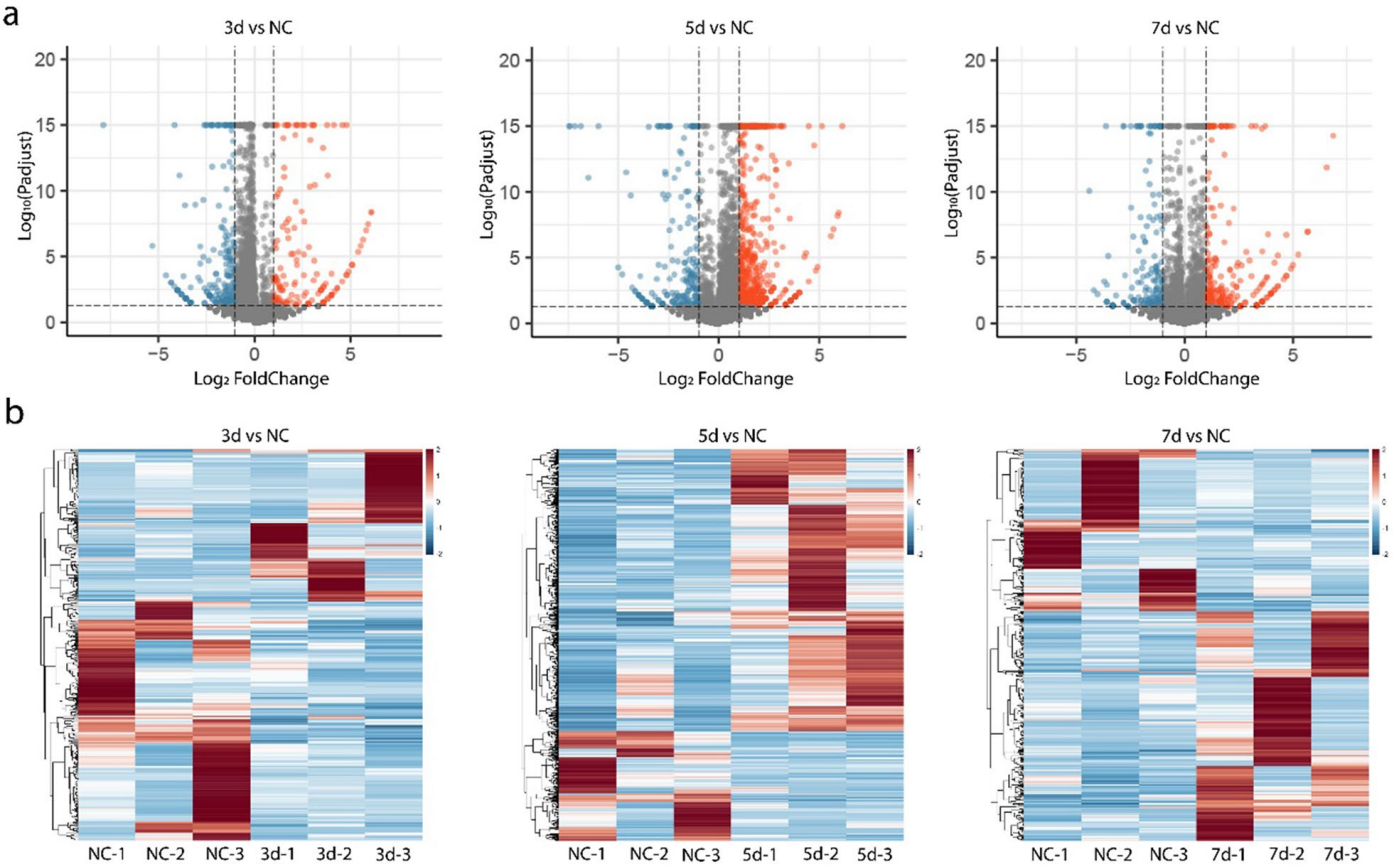
mRNAs expression profiles of SW2783 infected and NC groups. **a** Volcano map of DEGs, gray is the gene with non-significant difference, red and blue are the genes with significant difference (red represents up-regulated, blue represents down-regulated); The X-axis direction is the display of log_2_ Fold Change, and the Y-axis direction is − log_10_ (*P* adjust). **b** Hierarchical clustering Heatmap of DEGs, red represents upregulated and blue represents downregulated. DEMs, differentially expressed miRNAs; DEGs, differentially expressed genes; 3d, 3 days post infection; 5d, 5 days post infection; 7d, 7 days post infection; NC, negative control

Compared with the NC group, 372, 850 and 425 mRNAs were differentially expressed in the 3 dpi (138 up-regulated and 234 down-regulated), 5 dpi (568 up-regulated and 232 down-regulated), and 7 dpi (425 up-regulated and 174 down-regulated) groups, respectively. Based on the volcano map and heat map (Fig. 2), the DEGs were more up-regulated in the 5 dpi and 7 dpi groups, and more down-regulated in 3 dpi the group. Among them, most DEGs were found in the 5 dpi group. Differences in genes expression were observed at different stages of viral infection.

### Target gene prediction, GO and KEGG enrichment analysis and KEGG orthography (KO) analysis

**Fig. 3:**
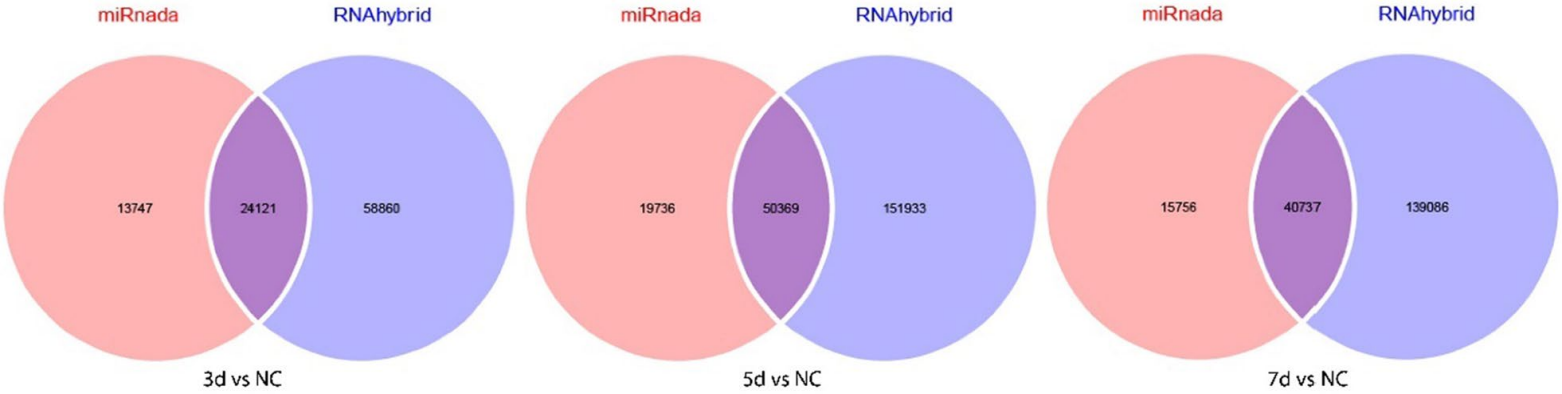
Venn Diagrams of target gene in two databases. Blue and red respectively represent the number of target genes predicted using the RNAhybrid and Miranda databases; 3d, 3 days post infection; 5d, 5 days post infection; 7d, 7 days post infection; NC, negative control

**Fig. 4:**
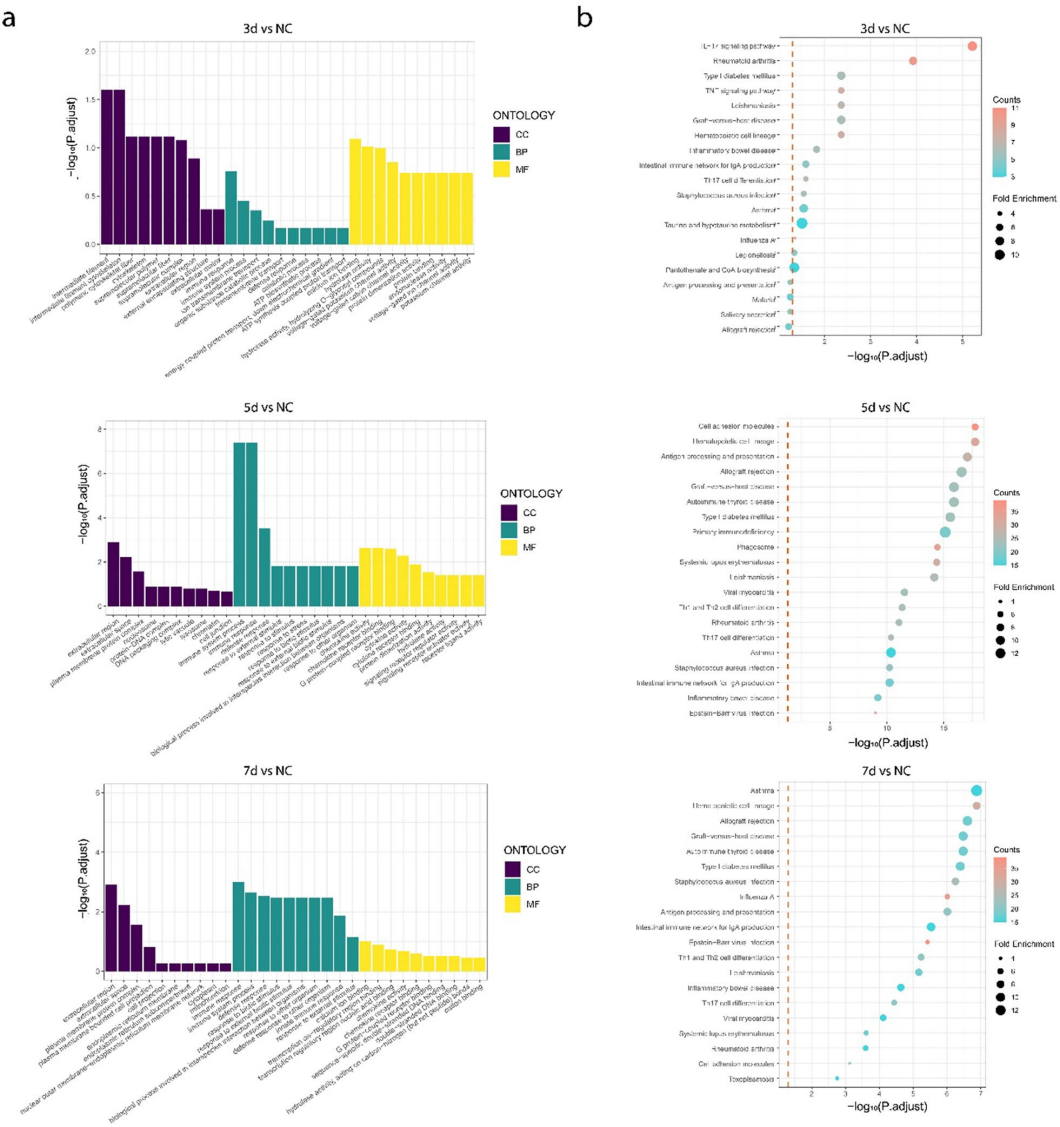
Go and KEGG enrichment maps of DEGs. **a** The picture shows the top ten entries (30 in total) in the three categories (biological process, cellular component, and molecular function) according to the adjust *P* value from large to small. Red, green, and blue represent the BP, CC, and MF, respectively. **b** According to the adjust *P* value and number of enriched mRNAs, the picture lists the top 20 pathway entries; the bubble color is red to blue, indicating that the adjust *P* value decreases, and the larger the bubble, the more mRNAs are enriched. GO, Gene Ontology; KEGG, Kyoto Encyclopedia of Genes and Genomes; DEMs, differentially expressed miRNAs; DEGs, differentially expressed genes; 3d, 3 days post infection; 5d, 5 days post infection; 7d, 7 days post infection; NC, negative control

RNAhybrid and miRanda databases were used to predict the target genes of the DEMs, and the intersection was obtained using a Venn diagram, as shown in Fig. 3. Among them, 24,226 target genes were predicted by two databases in 3 dpi VS NC, 42,181 in 5 dpi VS NC, and 37,956 in 7 dpi VS NC, as shown in Table 2. To elucidate the regulatory pathways and functions of DEMs in tree shrews during SW2783 infection, we cross-linked DEGs after viral infection with the target genes predicted by DEMs, and performed GO and KEGG enrichment analyses (Fig. 4). The results showed that 321 terms were enriched in the 3 dpi group, 330 terms in the 5 dpi group, and 197 terms in the 7 dpi group, with the highest enrichment in the 5 dpi group. The GO terms enriched by DEGs were basically the same, but the number differed at different times. In the classification of biological process (BP), the GO terms enriched were mainly related to cellular processes, biological regulation, stimulus response, immune system processes, localisation, and organisation or biogenesis of cellular components, such as, GPD1, BATF2, IRF1, CXCL10, RSG4, NEU2, etc. In cellular component (CC), the terms enriched including cell membranes, organelles, extracellular regions, polymer complexes, membrane-sealed chambers, and cell junctions, such as, C1QC, GNG7, PSMB8, CTSC, KRT4, PDK4, etc. In molecular function (MF), the most frequent functional changes in virus-infected tissue were related to binding, signaling, molecular transduction activity, catalytic activity, molecular function regulation, and transport activity, such as, CXCL9, IL1B, NHLH1, OTX2, S100A2, etc. The enrichment of immune response, stimulus response, and biological regulation increased significantly in 5 dpi and 7 dpi groups; However, the enrichment of cell components or tissues, development, and other processes increased significantly in 3 dpi group, and began to decrease at 5 dpi group, and decreased significantly or did not occur again at 7 dpi group, indicating that the antiviral response and function of damage repair gradually subsided and returned to normal with the control of infection at this stage. The enrichment of functions related to molecular transduction and catalytic activity after viruses infection increased significantly in the 5 dpi group, and decreased to a level similar to that in the 3 dpi by the 7 dpi group. Combined with CC, it was suggested that the viral reproductive activity is reduced and the virulence is weakened at this stage in vivo.

In the KEGG pathway enrichment analysis, 293, 303, and 284 pathways were enriched in the 3, 5 and 7 dpi groups, respectively. Among the pathways with the most significant post-infection enrichment, those related to the immune system, signal transduction, signal molecules and interactions, and viral infectious diseases were concentrated at various time points. These pathways were mainly involved in influenza virus infection and antiviral immunity; however, the number of major pathways and DEGs covered at different time points were different. The important pathways involved in the 3 dpi group mainly include haematopoietic cell lineage, cytokine-cytokine receptor interaction, IL-17, NF-κB and TNF signaling pathway. The altered genes inclusion of S100A9, IL6, CXCL8, CCL20, IL1B, CSF3 and TNFAIP3, indicating that in the early stage of infection, the host activates innate immune cells to produce immune effects and inflammatory responses in various ways, giving play to the role of early antiviral; In the 5 dpi group, the pathways included cell adhesion molecules, immune networks that produce IgA, haematopoietic cell lineages, antigen processing and presentation, Th17 cell differentiation, Th1 and Th2 cell differentiation, and phagosomes, such as, IL1B, CD14, IFIH1, CCL21, ICAM1, CD40, etc. At this time, the enrichment of DEGs in the above pathways increased significantly, indicating that the adaptive immune response was initiated and the functions of innate and adaptive immune cells were active, playing a strong antiviral role. In the 7 dpi group, the pathways included antigen processing and presentation, haematopoietic cell lineage, immune network-producing IgA, influenza A virus, Th1 and Th2 cell differentiation, Th17 cell differentiation, and cell adhesion molecules, including MX1, ISG15, IRF7, CXCL10, OAS2, etc. By this time, the DEGs had gradually decreased, indicating that the adaptive immune response was the main response at this stage. Under proper immune regulation, the immune effect gradually weakens and recovers to a steady state, indicating that the host is in the stage of infection recovery. Further analysis showed that among the significantly enriched KEGG pathways related to the immune system, most of the DEGs were up-regulated, especially in the 5 dpi group, which involved the innate and adaptive immune pathways, indicating that the host immune system played an effective immune effect after virus infection. Notably, IL-17 and Th17 cell pathways were significantly enriched after tree shrews infected with SW2783, suggesting that they may play an important role in regulating SW2783 infection and inflammatory response.

**Fig. 5:**
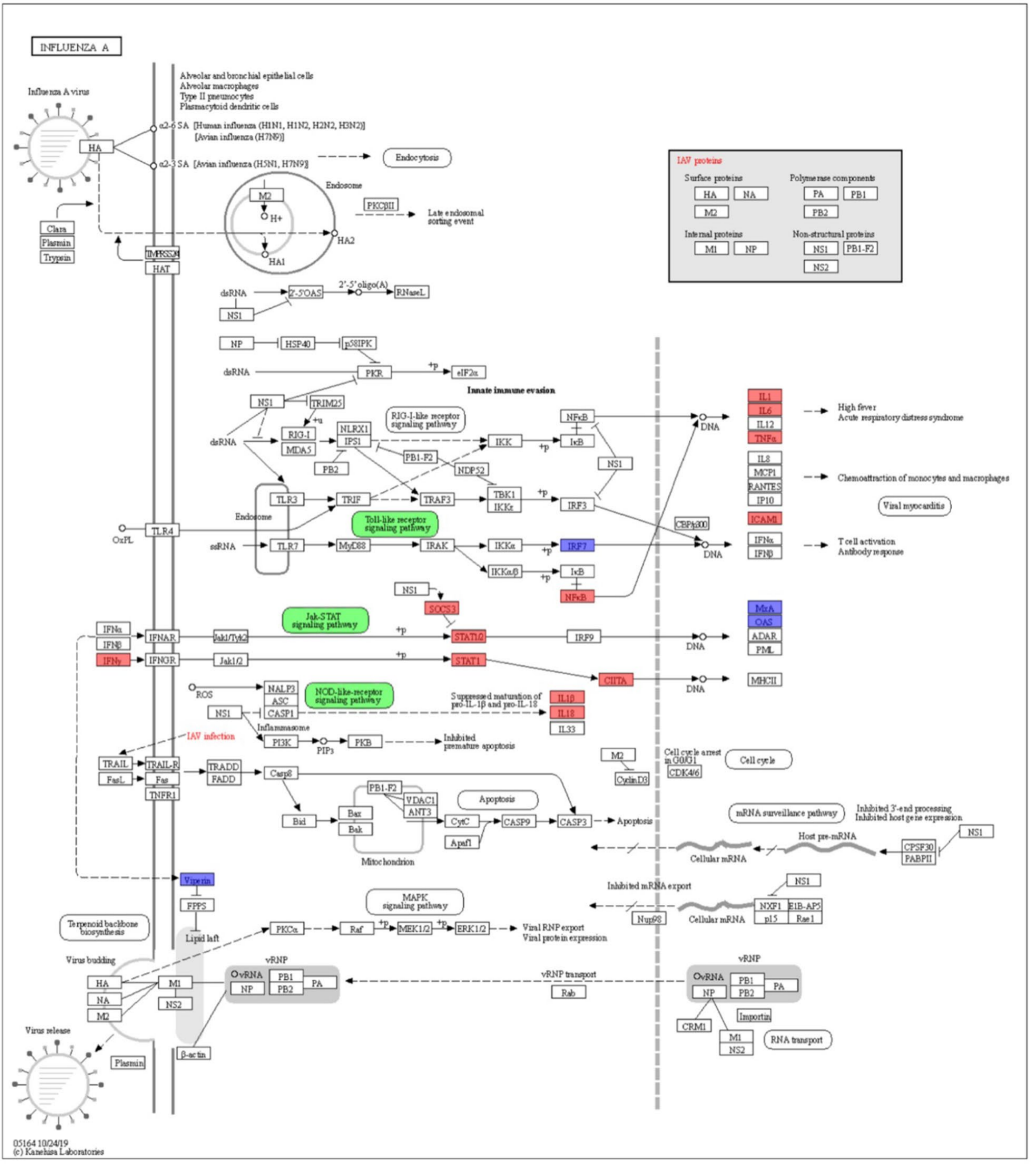
DEGs involved in the influenza A pathway (ko05164). Red represents high-expression genes, blue represents low-expression genes, and green represents signal pathways. DEGs, differentially expressed genes

According to the KEGG pathway enrichment analysis, the DEGs of SW2783 infection were analyzed and displayed in the IAV pathway (ko05164). The DEGs in any groups at the three time points after infection are shown in Fig. 5. It can be found that the DEGs are mainly concentrated in Toll-like receptor signaling pathway, NOD-like receptor signaling pathway and JAK-STAT signaling pathway, which are consistent with the results of KEGG functional enrichment. These show that some miRNAs negatively regulate gene expression in the immune response through changes in expression after SW2783 infection, to stimulate the immune function of the body and maintain the balance of the body against viral infection.

### Timeliness analysis

**Fig. 6:**
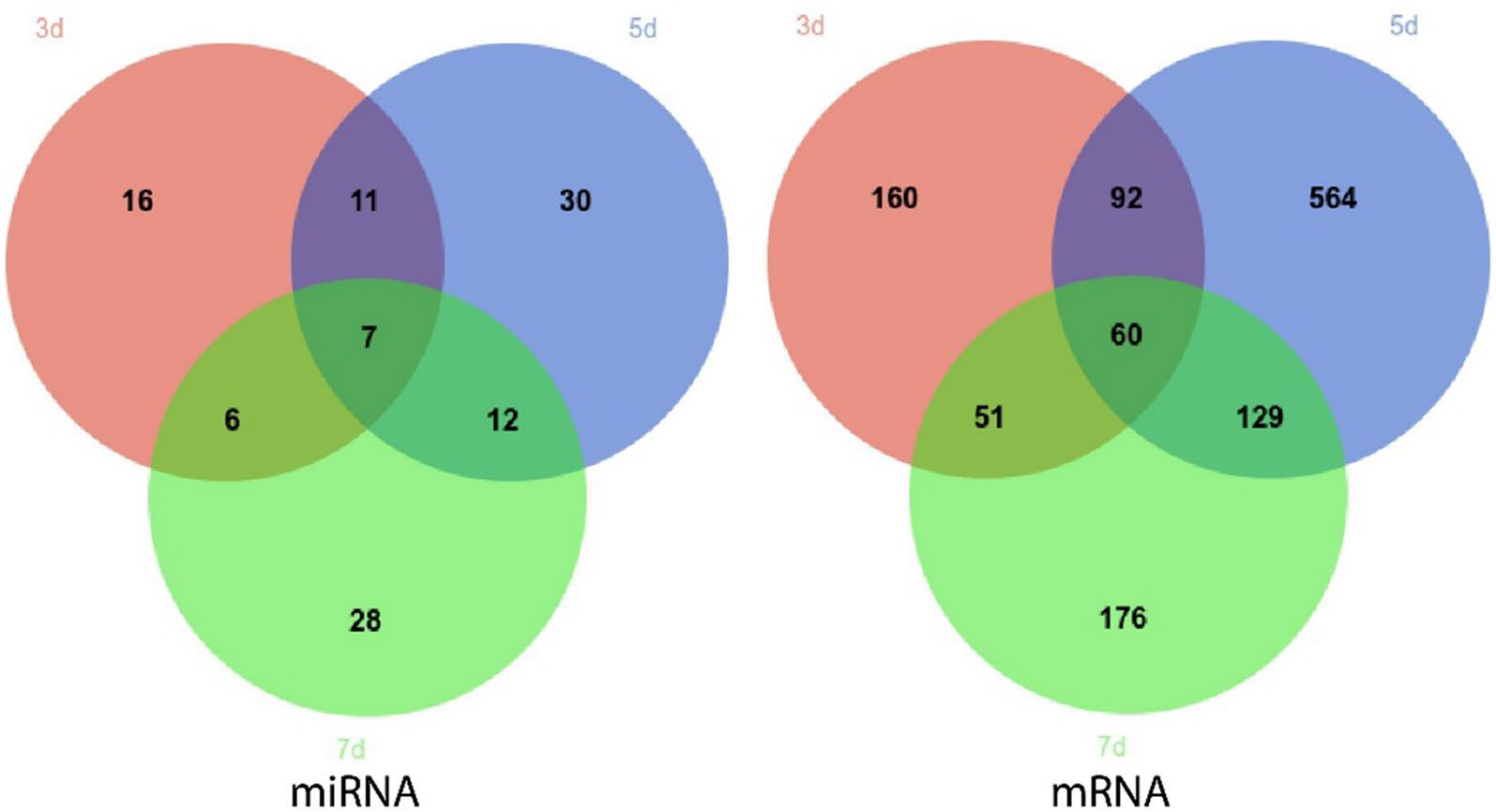
Venn diagrams of DEMs and DEGs on different days after infection. 3d vs NC (red), 5d vs NC (blue), 7d vs NC (green). DEMs, differentially expressed miRNAs; DEGs, differentially expressed genes; 3d, 3 days post infection; 5d, 5 days post infection; 7d, 7 days post infection; NC, negative control

Venn Diagrams of DEMs and DEGs on different days after SW2783 infection was conducted (Fig. 6). The top ten genes with differential expression multiples in each group are shown in Table 3. Among these, seven miRNAs and 60 mRNAs were differentially expressed at all three time points after infection. Combined with functional analysis, S100A8, S100A9, S100A12, and other genes were consistently highly expressed under the regulation of upstream miRNAs, suggesting that these miRNAs and mRNAs have a continuous impact on the host during the stage of virus replication and activation of immune regulation after early viral infection. It was also found that the expression of these three genes was the highest in the 3 dpi group and lowest the in the 7 dpi group, showing that with the response of the body to the virus, the body tended to be stable and the expression of the effector genes gradually recovered. There were 11 miRNAs and 92 miRNAs that were differentially expressed in both 3 dpi and 5 dpi groups, Among them, miR-324-3p, miR-3120 and others play a regulatory role in the early replication stage after viral infection by regulating genes such as FOSB, BCL3 and TNFAIP3, and the up-regulation of IL1B and CSF3 stimulate the body to induce innate immune response to resist virus infection, moreover, the expression levels of these genes in the 3 dpi and 5 dpi groups were basically the same, and decreased to the corresponding level with the NC group in the 7 dpi group, indicating that the body exerted innate immune response at the 3rd and 5th days after infection, and this effect disappeared at the 7th day. In addition, IL-6 was differentially expressed only in the 3 dpi group and CCL5 only in the 5 dpi group, which played a role in the signal transduction of antigen presentation and mediating the transformation from innate immunity to adaptive immunity. The expression of IFIT3, CCL21 and CCL15 increased at the same time as C1QB and IRF1, which played an innate immune response at the 5th day, with significant differences, affecting pro-inflammatory and apoptotic cell pathways and mediating the adaptive immune response of the host; 12 miRNAs and 129 mRNAs were differentially expressed in both 5 dpi and 7 dpi groups, miR-31, miR-16b, miR-106b-3p and many miRNAs changed significantly, which regulated C1QC, SELE, IFIT2, LTF, CD28 and others stimulates the body to produce an adaptive immune response while maintaining a fixed immune function and jointly resisting viral infection. In addition, the expression of the above genes reached a peak in the 5 dpi group, and lower in the 7 dpi group, showing that the immune defense function of the body showed a downward trend, and the body was gradually stabilizing. The expression of many genes involved in innate immunity was down regulated from 5 to 7 dpi group, and there was no significant difference compared with the NC group; By comparing the 3 dpi and 7 dpi groups, it was found that miR-429-1, miR-143-3p-1, etc. were differentially expressed in both two groups, and they mainly regulated CALCRL, MYO3B, SP7 and others, which played a role in the regulation of transcription and signal transduction, which may be related to cross-species infection of SW2783. However, in addition to the high expression of some genes related to adaptive immune function in the 7 dpi group, the expression of HSPH1, other heat shock proteins, and anti-inflammatory factors were upregulated to maintain the immune balance of the host. Differential expression was observed at different time points after SW2783 infection, indicating that there are different regulatory mechanisms at different stages of viral infection and the immune system, as well as the molecular regulatory mechanism of miRNA-mRNA after influenza virus infection is timeliness.

**Table 3:** The genes with the top 10 differential expression multiples

Group	miRNA	mRNA
3 dpi	miR-152-1, miR-122-5p, miR-93-5p, miR-199a-5p, novel_mir156, miR-30c, miR-135b, miR-103b, novel_mir67, miR-1301	DPYSL3, IL36G, SOX11, IL-6, CXCL8, TNFAIP2, SOCS3, EMX2, OLIG1, CCN3
5 dpi	miR-23b-3p-2, miR-107, miR-29a, miR-28-5p, miR-134-5p, miR-21-5p, miR-9-5p-1, miR-103-1, novel_mir128, novel_mir7	CCL15, CCL21, SOCS1, IFIH1, C1QB, STAT4, IRF1, CCL5, CXCL9, IFIT3, TLR2, SELE
7 dpi	miR-125b, miR-126-1, miR-200b-5p, miR-205-1, miR-199a-3p, miR-760-3p, miR-425-5p-1, miR-34a, miR-30e-3p, miR-26c-1	HSPH1, SPHKAP, SP7, SEMA7A, IFIT5, ISG20, IRF7, MX2, ISG15, PCDH10
3 dpi, 5 dpi	miR-191-5p, miR-3120, miR-409-3p-2, miR-9c-3p, miR-148a-3p, miR-34a-3p, miR-885, miR-324-3p, miR-148a-5p, miR-335	IL1B, CCL20, CSF3, BIRC3, BCL3, TNFAIP3, FOSB, SMAD9, GNG7, MUC4
5 dpi, 7 dpi	miR-449b, miR-106b-3p, miR-449a, novel_mir76, miR-425-5p-2, miR-31, novel_mir214, miR-16b, miR-218	CXCL11, CXCL13, CXCR3, CD3E, CD28, C1QC, TRIM22, IFIT2, MX1, LTF
3 dpi, 7 dpi	miR-26a, miR-143-3p, miR-429-1, miR-425-3p-4, novel_mir222, miR-100-5p	REM2, CHGB, LPO, CDYL2, STMN1, LY6D, PADI4, NEUROD1, ELAVL4, TEX15
3 dpi, 5 dpi, 7 dpi	miR-199b-2-5p, miR-3604, miR-126-3p-1, miR-429-2, miR-429, novel_mir259, miR-126-3p	S100A8, S100A9, S100A12, STMN2, GNG8, MYO3B, NHLH1, GAP43, ACTL6B, MEF2B

### Cross linking analysis of miRNA and mRNA

**Fig. 7:**
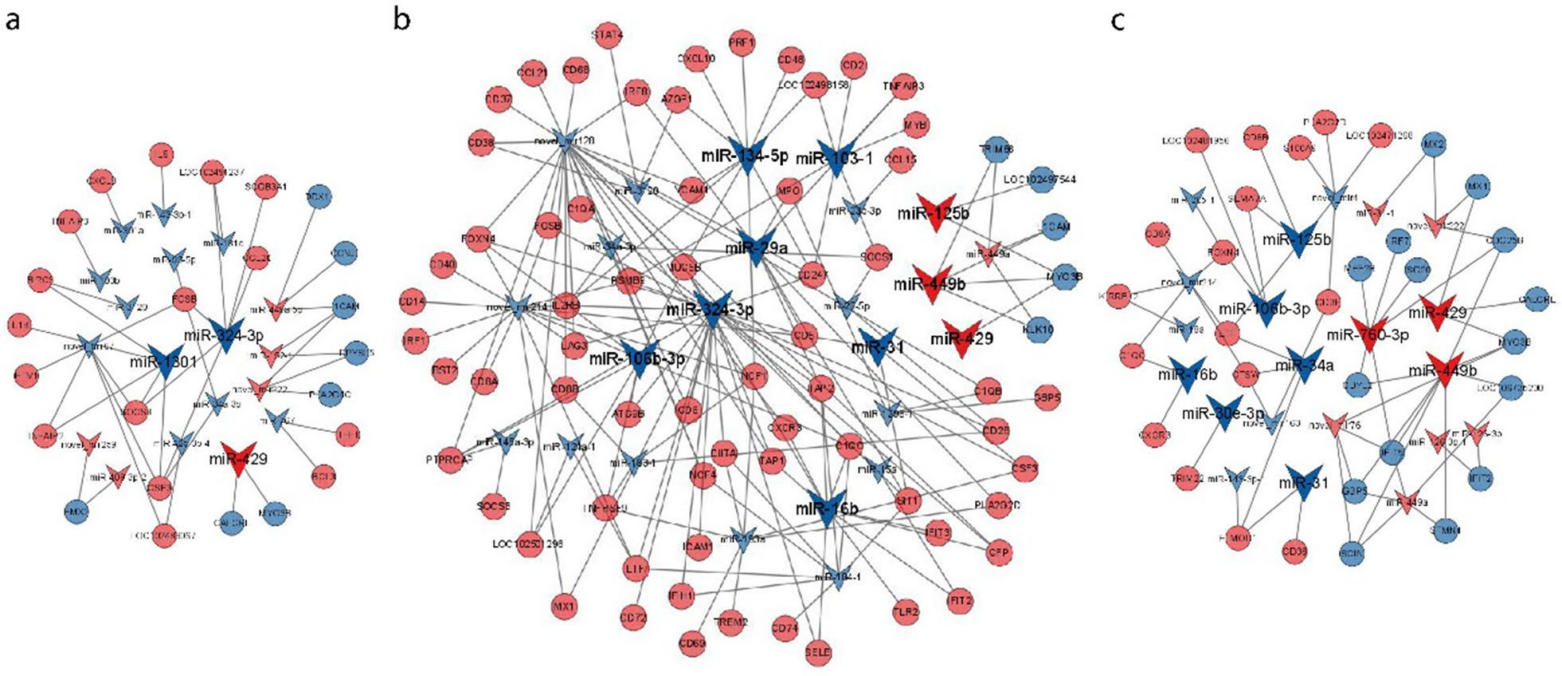
miRNA-mRNA relationship pairs involved in the regulation of SW2783 infection. **a** 3d vs NC. **b** 5d vs NC. **c** 7d vs NC. Arrows represent miRNA, circles represent mRNA, blue represents downregulated, and red represents upregulated; 3d, 3 days post infection; 5d, 5 days post infection; 7d, 7 days post infection; NC, negative control

The DEGs were intersected with the target genes predicted by DEMs through R, so that the screened DEGs and DEMs had targeting relationships. Cytoscape was used to create network maps of differentially expressed miRNA-mRNA interactions in the 3, 5 and 7 dpi group. There were 408, 1781, and 519 interaction pairs in each group. Due to the expression of many miRNAs and mRNAs was observed to change after SW2783 infection, we selected those only related to replication, antiviral, and immune negative regulation. These miRNA-mRNA interactions may be closely related to many signaling pathways and biological functions during SW2783 infection. After screening, 133, 553, and 132 interaction pairs were found to play a regulatory role in the process of infection in the 3, 5 and 7 dpi groups, respectively, as shown in Fig. 7.

Through the analysis of differentially expressed miRNA-mRNA interaction networks combined with the functional analysis of GO and KEGG, as well as the principle of miRNA-mRNA reverse regulation, the expression of miRNAs such as miR-1301, miR-324-3p, and novel_mir67 were significantly downregulated in the 3 dpi group, their target genes, such as MSLN, BARX2, FOSB, ETV1, and DPYSL5, played a regulatory role in the process of viral replication; and IL1B, CCL20, TNFAIP2, CSF3, GNG8, BIRC3, SOCS3, and many others were up-regulated, which played a role in immune response. miR-103-1, miR-134-5p-1, miR-16b, and novel_mir128 were significantly down-regulated, and miR-449b was up-regulated in the 5 dpi group. MYB, MUC5B, FOXN4, TFEC, and others were involved in viral replication and transcription. RSG4, IFIT2, IFIT3, TLR2, PSMB9, TNFAIP3, SOCS1, C1QB, C1QC, AZGP1, SIT1, and CXCL10 were involved in innate immune signaling pathways and CD2, CD38, CD48, CD74, IL2RB, NCF1, CSF3, LOC102498158, CCL21, IRF8, and SELE were involved in adaptive immunity. The expression of miR-106b-3p, miR-34a, and novel_mir214 was down-regulated, whereas that of miR-760-3p was up-regulated in the 7 dpi group. Their target genes, such as INSM1, LPP, FOXN4, TRIM22, CDYL2 and SP7, played a role in signal transduction during multiplication, the expression of SEMA7A, LOC102481956, LTF, CTSW, CD8A, C1QC, etc. are up-regulated to activate immune system, while the expression of IRF7, IFIT5, ISG20, etc. are down-regulated to play a negative regulatory role in immune response. The targeting relationship between the miRNAs and mRNAs screened above is shown in Table 4.

**Table 4:** Relationships between miRNAs and inversely correlated target genes

miR_name	Regulation	Target gene	Regulation
miR-324-3p	Down	TNFAIP2, MX1, IFIH1, CCL20, LTF, SELE	Up
miR-1301	Down	IL1B, GNG8, TNFAIP2, MSLN, CSF3, BARX2	Up
miR-106b-3p	Down	KLK1, FOXN4, MUC4, CD163, TFEC	Up
miR-103-1	Down	RSG4, SOCS1, TNFAIP3, LOC102494593	Up
miR-134-5p	Down	AZGP1, CD48, IL2RB, NCF1, LOC102498158	Up
miR-29a	Down	IRF8, TNFAIP3, NLRC5, SIT1	Up
miR-31	Down	SIT1, CSF3, NLRC5, NHLH1, CD5	Up
miR-760-3p	Up	IRF7, CDC25B, DPYSL5, IFIT5, MEF2B	Down
miR-16b	Down	IFIT2, TLR2, C1QC, SELE	Up
miR-34a	Down	LTF, SP7, CTSW	Up
miR-449b	Up	MYO3B, TRIM66, RRM2, CRMP1, KIF1A	Down
miR-125b	Down	CD8B, TLR2, NLRC5, SEMA7A	Up
miR-429	Up	CALCRL, MYO3B, MX1	Down
miR-30e-3p	Down	TRIM22	Up

### Preliminary verification of DEMs and DEGs by RT-qPCR

**Fig. 8:**
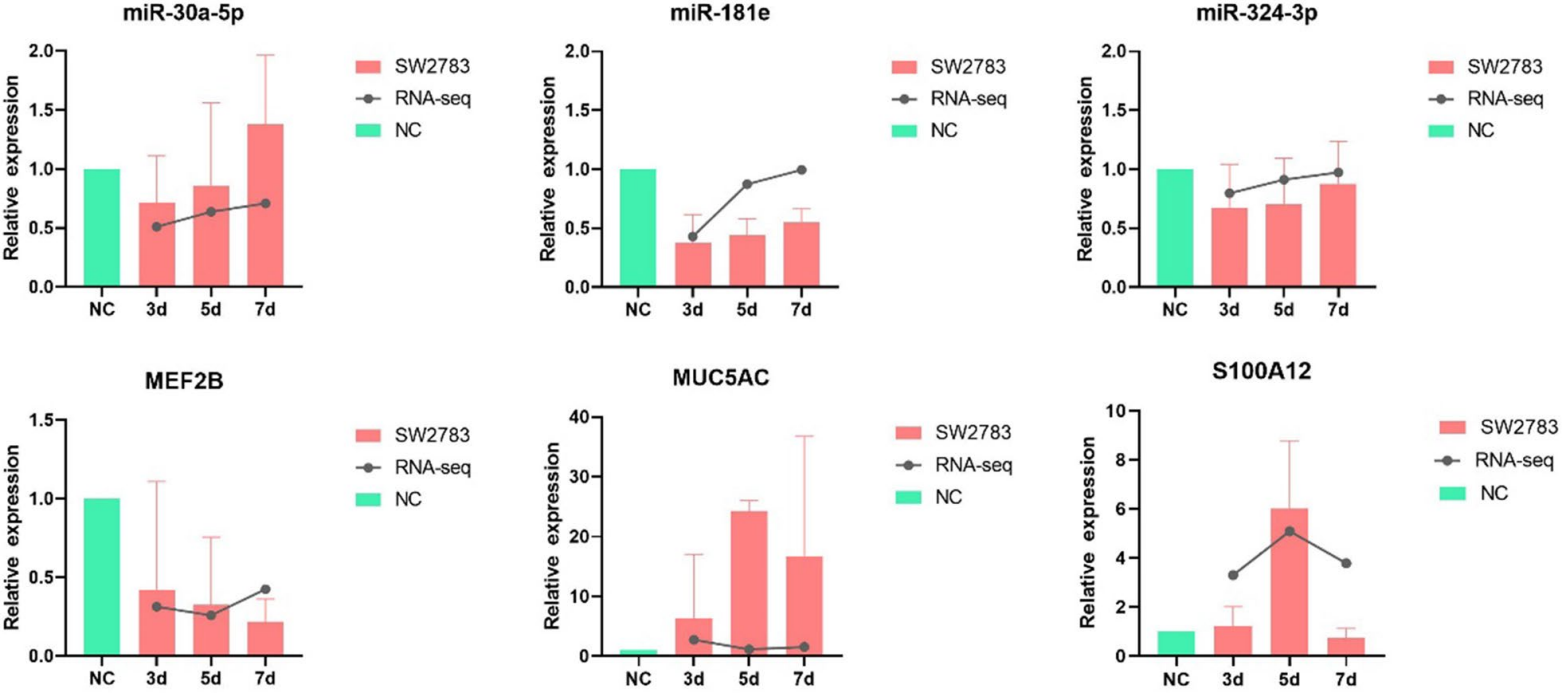
RT-qPCR validation of selected DEMs and DEGs. DEMs, differentially expressed miRNAs; DEGs, differentially expressed genes; 3d, 3 days post infection; 5d, 5 days post infection; 7d, 7 days post infection; NC, negative control

The miRNAs and mRNAs obtained by sequencing were verified using RT-qPCR to ensure the credibility and accuracy of the RNA-seq. We verified three miRNAs (miR-30a-3p, miR-324-3p, and miR-181e) and three mRNAs (S100A12, CD74, and MEF2B), respectively. And the results showed that the expression trends of the DEMs and DEGs in RT-qPCR were consistent with the sequencing results (Fig. 8), indicating that the RNA-seq results were accurate and reliable.

### Validation of interaction networks

**Fig. 9:**
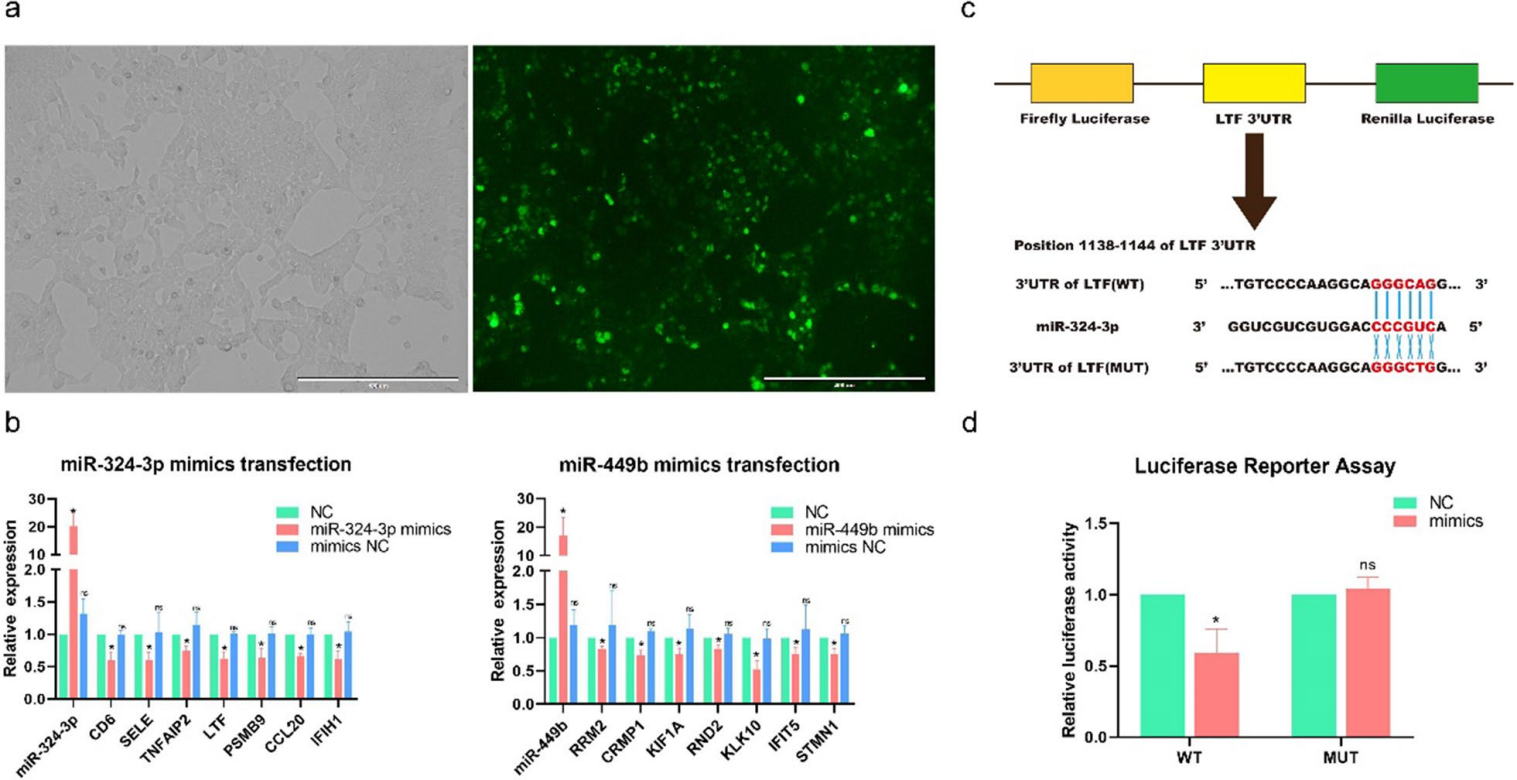
Verification of interaction networks. **a** The transfection efficiency was observed by fluorescence microscopy. **b** Expression levels changes after miRNA mimics transfection. The overexpression effect of miRNA mimics in 293 T cells was used to detect miRNA and mRNA expression levels 48 h after transfection by RT-qPCR. One-way ANOVA with Dunnett’s multiple comparison test. Means ± SD, compared with the NC group. The experiments were performed at least three times independently. **c** Schematic drawing of the putative binding sites or mutations of miR-324-3p in LTF mRNA 3′ UTR. **d** Luciferase activity of each group was detected at 48 h post-transfection, compared with the NC group, *P < 0.05; ns, no significance

To validate the regulatory relationship between miRNAs and mRNAs expression in the interaction network, we investigated the effect of changes in the expression of two representative miRNAs (miR-324-3p and miR-449b) of tree shrews infected with SW2783 on the expression levels of their target genes (miR-324-3p: LTF, CD6, SELE, TNFAIP2; miR-449b: RND2, RRM2, CAMP1, KIF1A). We designed and transfected two specific miRNA mimics into 293 T cells to enhance miRNA expression, and then detected the expression of miRNA and their target genes by RT-qPCR. The results showed that miR-324-3p and miR-449b were significantly upregulated after transfection, and each target gene had varying degrees of decreased expression in the transfection group, with statistical significance compared with the NC group (*P* < 0.05), as shown in Fig. 9b. The results indicated that there was a targeted negative regulatory relationship between screened miRNAs and mRNAs by differential and functional analysis. Based on the above results, luciferase reporter assay was continued to verify the targeting relationship between miRNAs and mRNAs in the network, and miR-324-3p and LTF were selected to prove the existence of miRNA-mRNA regulatory axis during viral infection. Firstly, the binding sites of miR-324-3p and LTF were identified (Fig. 9c). After co-transfection with miR-324-3p mimics and WT plasmids, the luciferase activity was significantly inhibited, decreased by about 50%, and the difference was statistically significant (*P* < 0.05), as shown in Fig. 9d. The results showed that there was a direct targeting relationship between miR-324-3p and LTF, which verified the accuracy of the above target gene prediction and the construction of the virus-related miRNA-mRNA interaction network, indicating that the expression of target genes could be regulated by adjusting the screened miRNAs, forming the miRNA–mRNA regulatory axis to play the role of antiviral infection.

## Discussion

After IAVs cross-species invade the host, they affect the genes expression and proteins synthesis of the host during transcription and replication and can stimulate the inflammatory and immune response or cause pathological damage to the host [26]. MiRNAs play an indispensable role in the regulation of biological activities in nature. Their regulation is usually not isolated, but constitutes a regulatory network through a coordinated mechanism, and a miRNA can simultaneously target multiple genes located in the same signaling pathway, which is widely used in clinical medicine [27–29]. In addition, many studies have demonstrated that some miRNAs can inhibit IAVs replication and exert antiviral effects by targeting the expression of PB1, PB2, M1, NA, and other viral genes in the host [30]; There are also miR-20a-5p and miR-29c to resist the invasion of influenza virus by regulating immune-related genes, such as cytokines and chemokines, or enhancing the ability of presenting disease antigens [31–33]. However, there are relatively few studies aimed at studying the effect of miRNAs on influenza virus cross-species infection by regulating the signal pathway through target genes expression. Therefore, strengthening the study of miRNAs expression and its possible regulatory signaling pathway in the immune system after IAVs infection is of great significance for influenza virus prevention and treatment, new targets for vaccine research and cross-species infection prediction.

Our preliminary study indicate that tree shrews can better simulate the pathogenicity, transmissibility and immunity of human influenza virus infection, and are suitable mammalian models for studying the pathogenicity and cross-species transmission of IAVs. The results showed that the newly emerged SIV SW2783 can infect tree shrews and cause discomfort, and has strong infectivity and replication activity in the respiratory tract tissue of tree shrews, mainly causing self-limited upper respiratory tract infection in turbinate tissue [9]. Consistent with the viral replication symptoms and clinical symptoms phenotype, with the enhancement of viral replication, the host initiates the corresponding natural immune response, which plays an important role in antiviral activity [9]. Therefore, in this study, tree shrews were selected as the animal model of newly emerged SIV infection, the turbinate tissues were sequenced, and the miRNA and mRNA expression profiles were comprehensively analyzed at 3, 5 and 7 dpi with the NC group, so as to explore the characteristics of miRNAs and mRNAs changes in respiratory tissue cells of cross-species infected tree shrews at different time points. To investigate whether there are specific DEMs that play a key role in the process of cross-species infection and host interaction of the influenza virus, analyzing and predicting the key regulated target genes and their possible mechanisms of action through bioinformatics software, and preliminarily verifying the accuracy of the analysis.

The gradual adaptation of IAVs to the host and its ability to continuously break through species barriers, including the ability to bind to and enter host cells, replicate within host cells, evade host limiting factors and innate immune responses, and spread between host individuals, are key to their ability to obtain cross-species transmission and cause a new host pandemic. The ability of virus replication and immune escape is particularly important in the process of cross-species transmission. Therefore, based on the previous successful establishment of a model of cross-species infection of low pathogenic animal IAVs in human respiratory tract tissues and tree shrews without adapted, this study intended to conduct integrated analysis of expression profiles in turbinate tissues of tree shrews infected with SW2783. Through integrated analysis of sequencing results, a total of 40, 60, and 53 DEMs, 372, 850, and 425 DEGs were selected in the 3, 5, and 7 dpi groups, respectively. In addition, 408, 1781 and 519 miRNA–mRNA interaction pairs were constructed at three groups by bioinformatics tools prediction and analysis, among which 133, 553 and 132 may play a regulatory role in SW2783 infection of tree shrews. Combined with functional analysis, miR-760-3p, miR-449b, miR-30e-3p and miR-429 may play an important role in the regulation of viral replication; miR-324-3p, miR-1301, miR-103-1, miR-134-5p, miR-29a, miR-31, miR-16b, miR-34a, and miR-125b may regulate the immune system to defend against virus infection; It is speculated that miR-106b-3p may be related to the cross-species infection potential of SW2783.

We have reviewed a large number of relevant literatures, and most of the results show that the changes in miRNAs are different after IAVs infection, which may be species-specific, but the regulated target genes are partially similar. For example, in studies on infection with IAV in rhesus monkeys, chickens, and swine, the same miRNAs as ours were not found, but some target genes such as TNFAIP2, STAT3, FOSB, etc. were altered, mainly regulating inflammation, immune response, as well as MAPK, NF-κB anti-virus signaling pathways, similar to the results of our study [31, 34–36]. In addition, homologous IAVs were used in the study of chickens and swine, which is different from the cross-species infection in this study. Interestingly, in our previous study of cross-species infection of MDCK and human A549 cells with SIVs, no similar miRNA changes were observed. However, the changes in genes such as CXCL10, MX2, and IFIT2 were the same. It suggests that although miRNA changes are different, they may exert antiviral effects by regulating the expression of the same genes [2, 4]; In addition, in a study of microarray analysis after human nasal epithelial cells (hNECs) infection with human IAVs of H3N2 subtype, although identical DEMs were not found, more DEGs, such as IRF7, STAT1, CXCL10, and IL6, were found mainly triggered inflammatory reactions and innate immune responses in the transmission of signals such as interferon, JAK-STAT, and Toll like receptors, similar to the changes observed in our experiment [37]. The nasal turbinate tissue and hNECs are both from the upper respiratory tract, which makes the results more comparable. At the same time, it also indicates that the genome of tree shrews has high similarity to that of humans, making it a better animal model for studying influenza virus infection. These cases lead us to speculate that the possible reasons for different DEMs are differences in virus sources, whether cross-species infections, in vitro and in vivo experiments, different tissues or cells of the respiratory tract, analysis methods, and other aspects. However, the most critical factor may be that there are species differences among the experimental models, so the function of the miRNAs screened in this study to regulate influenza virus may be specific to tree shrews.

The outcome of IAVs infection depends on complex interactions between the hosts, viruses, and environmental factors [38]. The miRNA expression profiles of the host changed significantly after the IAVs infection. Previous research has shown that miRNA affects IAVs infection mainly through two ways: limiting virus replication and regulating immune response [37, 39–43]. Analysis of the expression profiles in our study revealed that miR-449a and miR-449b were highly expressed at the early stage of infection, whereas the expression of their target genes L1CAM, MYO3B, SCIN, and STMN1 decreased. MiR-449b has been proven to be a regulator of histone deacetylase 1 and interferon beta in IAVs infection [44]. Existing studies have shown that STMN1 plays an important role in viruses infection, and low expression of STMN1 inhibits dengue viruses replication [45, 46]; In addition, miR-760-3p regulates decreased expression of MEF2B and CDC25B. Some researchers have also verified the reduced replication of IAVs in CDC25B-knockout cells [47]. Combined with the results of these two studies, it is speculated that the increased expression of miR-760-3p and miR-449b may restrict the replication of SW2783 cross-species infection. Tripartite motif-containing (TRIM) proteins play an important role in the antiviral process. The results of this study found that the expression of TRIM22 increased under the regulation of miR-30e-3p. Functional analysis revealed that it was enriched in transcriptional regulation, signal transduction and immune regulatory-related pathways to play an antiviral role by inhibiting the transcription and replication of viruses at the early stage of infection. In line with previous studies by Isabel Pagani, TRIM22 has a limiting effect on the replication of different DNA and RNA viruses, especially in IAVs, controlling the transcription of viral genes, and TRIM22 has a pre-existing intracellular defense against IAVs infection in cells from the respiratory tract [48, 49]. In addition, it has been proven that miR-30e-3p inhibits the replication of influenza B virus by targeting virus NA and NP genes [40]. Therefore, through screening and analysis of the above expression profiles, miR-449b, miR-760-3p and miR-30e-3p may inhibit the transcription and translation of viral genes to prevent viral reproduction through the regulation of signal transduction in the early stage of SW2783 infection. It is speculated that the regulation mechanism of transcriptional signals may be related to the cross-species infective potential of SW2783 and have the potential to become the prevention targets of animal influenza viruses cross-species infected humans.

**Fig. 10:**
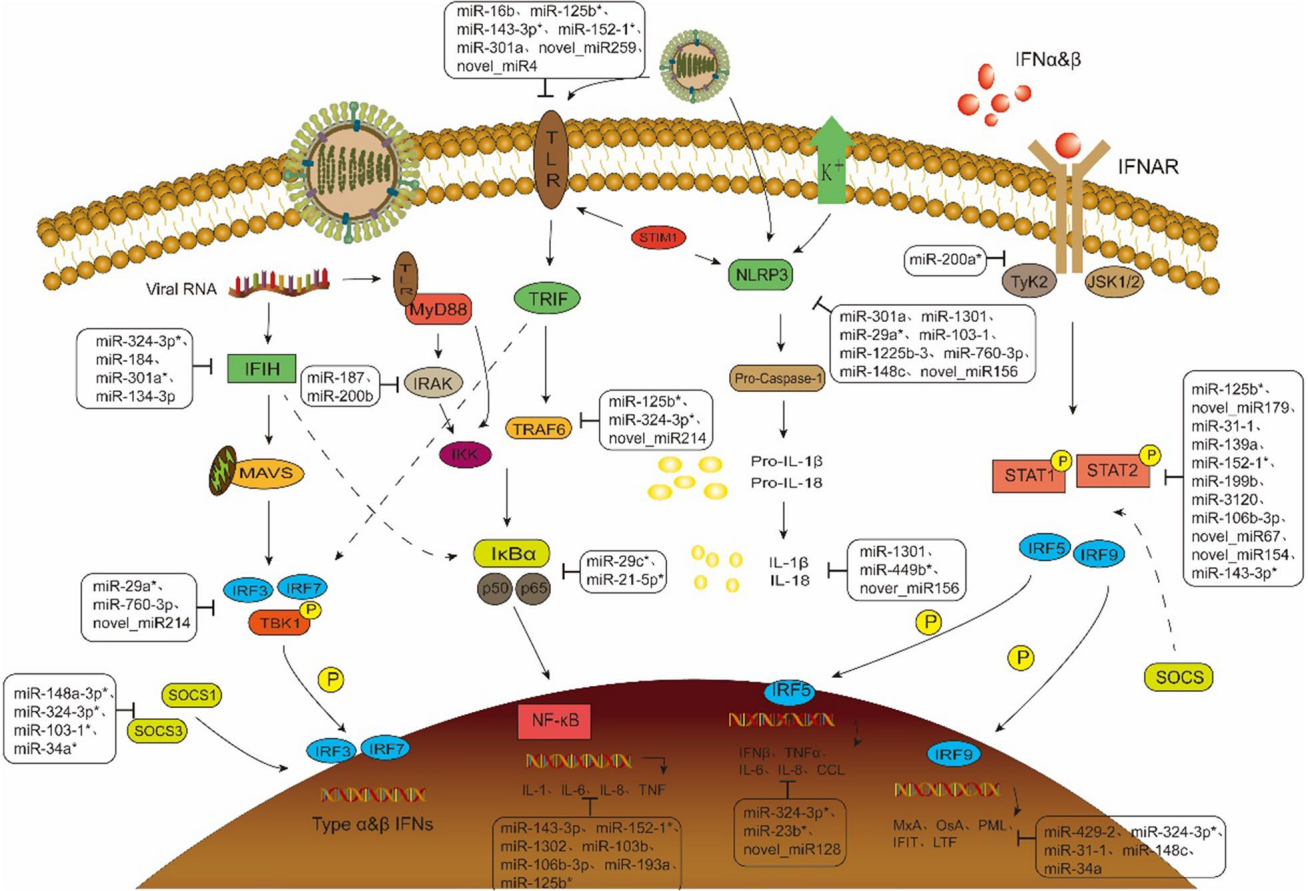
The pattern recognition and downstream signal pathway of influenza virus infection regulated by miRNA. Solid arrows indicate direct effects and dotted arrows indicate indirect effects. *Indicates that the miRNA has been reported to be involved in IAVs regulation [54–61]. The unlabeled miRNAs were selected in this study

The innate immune system is the first defense barrier against exogenous microbial infection. It is rapidly activated by recognizing pathogen-associated molecular patterns (PAMPs) in pathogens through pattern recognition receptors (PRRs) to detect infection. And then stimulate and activate a series of signaling pathways, such as NF-κB and IRF, induce the expression of pro-inflammatory cytokines, chemokines and type I interferon (IFN-α/β), and activate IFN-stimulated genes (ISGs) to produce a variety of antiviral proteins [50]. The screened miRNAs involved in the pattern recognition and downstream signaling pathways of influenza viruses infection are shown in Fig. 10. KEGG analysis showed that multiple signaling pathways of innate immunity were significantly enriched after IAVs infection, including NF-κB, chemokine, cytokine-cytokine receptor interaction, and toll-like receptor signaling pathways. Among them, miR-184, miR-324-3p, miR-16b, miR-125b, miR-155, miR-29a, and miR-31 were down-regulated, and significantly increased IFIH1, TLR2, and NLRC5. Recognizing the viruses and activating a series of signaling pathways play important roles in anti-influenza immune regulation, suggesting that these three types of PRRs signaling pathways are indispensable in tree shrews' resistance to SW2783 infection. It has been confirmed that miR-155 can target the expression of toll-like receptors and is extremely sensitive to tumor necrosis factor and interferon, which confirms the reliability of our results [51]. Therefore, in the prevention and treatment of IAVs, the expression of three PRRs can be promoted by regulating the expression of miRNAs, so as to identify the invading virus more sensitively to activate the immune response. In the analysis of the NF-κB signaling pathway, it was found that the up-regulated expression of miR-29c and miR-21-5p inhibited the expression of IκB, thus increasing the activity of the NF-κB signaling pathway, producing a variety of interleukins and tumor necrosis factors, and causing inflammatory effects to kill the influenza viruses. Studies have shown that miR-29c can reduce the activity of NF-κB and express TNF-α, INF-β, IL-6, IL-1b, and IL-8 through ubiquitinase A20 [52]. In addition, miR-3120 and miR-125b-3 were found in the interferon receptor signaling pathway to affect the expression of STAT and IRF, promoting the expression of cytokines, such as interleukins and chemokines, and increasing the body's killing effect on viruses. IRF5 can regulate the production of IFN-β, TNF-α, IL-6, IL-8, CCL2, and CCL5 after H1N1 infection, which lays the foundation for the inference of our results [53]. After influenza virus infection, the influence of DEMs and DEGs on a variety of signaling pathways was explored to provide scientific basis for further discussion of the prevention and control mechanism of influenza virus infection, evaluation of the effect of intervention strategies, and prediction of cross-species transmission potential.

On the other hand, miRNAs can also activate innate immune cells and regulate the activation, proliferation and differentiation of T and B cells in adaptive immune by affecting the expression of certain cytokines after IAVs infection, playing an important antiviral role [62]. In this study, miR-1301, miR-143-3p-1, miR-103b, and other miRNAs were down-regulated in the 3d group, and their targeted regulation of inflammatory cytokines such as IL-6 and TNFAIP2 was up-regulated to varying degrees. A large number of DEMs were added in the 5d group, such as miR-134-5p-1, miR-103-1, miR-125b, miR-324-3p, miR-193a, miR-16b, novel_mir214, and novel_mir128. Regulating various transcription factors, chemokines, adhesion molecules, and other inflammatory factors were significantly up-regulated, which are of great significance in innate and adaptive immune responses after IAVs infection. Differential expression of these miRNAs plays an important role in the cross-species transmission of SW2783. Simultaneously, miR-301a-CXCL8, miR-134-5p-CXCL10, miR-324-3p-CCL20, and miR-23b-3p-2-CCL15 regulate the expression of chemokines and promote migration, recruitment, differentiation, and activation of various innate and adaptive immune cells, which are indispensable for immune response [63–65]. The expression of most antiviral and inflammation-related miRNA–mRNAs in our study was also similar to the transcriptomic study by Tan, which used human influenza virus H3N2 to infect hNECs. The results showed that the nasal epithelial response was strongly inclined to the type I inflammatory response after viral infection. The JAK-STAT-mediated type I inflammatory response was activated by triggering the innate and adaptive immune activating cytokines IL-6, CXCL10, and IFNλs, which were similar to the results of our study, and the tree shrew turbinate tissue cells selected made the results more comparable [37].

To determine the changes in miRNAs expression at different stages after infection and the role of the regulatory mechanism by observing the differential expression in different time groups, which could provide a new target for precise regulation of influenza virus infection. In this study, we observed changes in miRNAs over time at 3, 5 and 7 days after tree shrews infected with SW2783. 7 miRNAs, including miR-429, miR-126-3p, and others, were highly expressed at the three time points after infection. By regulating the expression of CALCRL, MYO3B, DNASE1, FGF20, and other genes, they can inhibit intercellular signal transduction, transcription and replication processes to resist virus invasion and cell apoptosis after infection, and exert the immune response effect of interferon effector genes such as MX1, IFIT2, and CCL15. miR-324-3p showed significantly lower expression in the 3 dpi and 5 dpi groups, and the change in expression was not statistically significant in the 7 dpi group. Based on interaction analysis, miR-324-3p mainly regulated FOSB, MX1, and IFIH1, which can inhibit transcription and replication in the early stages of viral infection, and stimulate to produce the innate immune response. With the control of the infection on 7th day, the replication of the virus was reduced, and the innate immune function gradually faded back to normal. It has been indicated that miR-324-3p can regulate the expression of TRIM27 to reduce viral replication during infection, and TRIM27 is a negative regulator of IFN-α/β to induce antiviral immune response [66, 67]. The expression of miR-16b and miR-15a in the 3 dpi group were similar to those in the NC group, and began to be significantly down-regulated from 5 to 7th days. They regulated IFIT2, IFIT3, SELE, CXCR3, C1QC, and other genes to exert adaptive immune functions. The chemokine CXCL10 and interferon regulatory factors, such as IFIT2, ISG20, and IRF7, were down-expressed in the 7 dpi group under the condition of high expression of miR-126-3p and miR-760-3p, which changed from higher to lower than the control or no difference in the 5 dpi group. It could be that the response state of the body is relatively stable after 7 days, and the immune response is reduced to maintain the physiological balance of the body. Interestingly, although the expression of type I interferon (IFN-α/β) was not up-regulated on the 3rd, 5th and 7th days after infection, some miRNAs were down-regulated on the 5th day, including miR-16b, miR-324-3p, miR-124a-1 and miR-31, etc. ISGs expression was significantly up-regulated, including IFIT2, IFIT3, RSAD2, Mx1, CSF3, and LTF, suggesting that the IFN-α/β production pathway was activated and ISGs protein production was induced by above miRNAs. The results of our study were similar to those of Pont, who observed the infection of swine influenza virus H1N1 subtype in pig lung tissue and speculated that the reason might be that the induction of IFN-α/β occurred earlier than the 3rd day [68]. In summary, the expression profiles of miRNAs changes over time after IAVs infection, and they regulate downstream target genes changes over time and play different regulatory roles at different periods after infection. Therefore, the screening of miRNAs with different effects in different periods can lay a theoretical foundation for the research and development of influenza virus vaccines and drugs.

Because there are numerous subtypes of IAVs in nature and their high susceptibility to mutation, when a variety of IAVs are mixed with infection, genetic recombination occurs, resulting in increased virulence of the viruses and changes in the infected hosts, thus giving some IAVs the ability to infect cross-species. Some IAVs can overcome the species barrier and jump directly to humans under certain conditions [69]. Humans can be infected with avian influenza viruses (AIVs) H5N1, H6N1, H7N9, H9N2, H5N8, and H10N3, etc., and with SIVs H1N1, H2N2, and H3N2, etc. have been reported recently [70–77]. Therefore, the exploration of miRNAs that can interfere with cross-species transmission is of great practical significance for dealing with the threat of animal-derived influenza to humans. In this study, the emerging H3N2 strain SW2783, which recombination internal gene fragment of pdm/09 H1N1 had previously been verified to have cross-species infection potential, was selected for the integrated analysis of miRNA–mRNA after SIV cross-species infection. The results showed that the newly emerged SW2783 could not only infect tree shrews and cause tissue lesions, but also directly spread from tree shrews to guinea pigs across the species barrier [9], and could infect human lung tissue in vitro without adaptation (unpublished data). Through the integrated analysis of miRNA-mRNA expression profiles, it was found that the expression of FOXN4, MUC4, KLK1 and CD163 were up-regulated under the action of miR-106b-3p after infection. GO and KEGG functional analyses revealed that these DEGs mainly played the functions of transcriptional regulation and cell matrix attachment. FOXN4, a member of the forkhead box/winged-helix transcription factor superfamily, is an important transcriptional regulatory gene and significantly overexpressed in EBV-positive nasopharyngeal carcinoma (NPC), promoting EBVs replication and cells tumorigenesis [78]; KLK1 is a widely distributed serine protease, and it has been shown that the levels of KLK1 increased, in the lungs ofmice during influenza virus infection. KLK1 cleaved H1, H2, and H3 HA molecules and consequently enhanced viral production [79]; CD163 is the most important and critical molecule in the life cycle of Porcine reproductive and respiratory syndrome virus (PRRSV), which is responsible for mediating virus uncoating and genome release, so by up regating the expression of CD163, it promotes the replication of the viruses in the cells and thus increases the efficacy of infection [80]. MiR-106b-3p, as regulatory factors of the above mRNAs, decreased after infection, and combined with the these genes can promote viral replication to enhance infectibility, it was speculated that miR-106b-3p might be related to the cross-species infection potential of SW2783. However, there are few relevant studies on miR-106b-3p in influenza viruses, so further researches are needed to determine whether they are related to the cross-species infection potential of IAVs. In a study by Gao [4], SW2783, the same SIV as in this study, was selected to cross-species infect human A549 cells. 24 h after infection, miRNAs such as miR-21-5p and miR-29b-1-5p were under-expressed, and genes expression such as SCARB2, TRIM27 and RPS28 were up-regulated. Through functional analysis, it was found that they were mainly enriched in viral transcription, signal transduction, metabolism and others, which proved the potential of cross-species infection of SW2783. The functional analysis results of the above genes were similar to those of this study, suggesting that the expression changes of miR-106b-3p may also be related to the cross-species infection of IAVs. However, the above DEMs and DEGs could not be found in our study, which may be caused by the different establishment of the experimental models and time nodes. Nevertheless, these results were consistent with the cross-species infection ability of SW2783 previously confirmed.

## Conclusions

In conclusion the upper respiratory tract is the first portal invaded by the influenza virus and is the main site of virus replication. It is also the origin of the host antiviral response to IAVs infection and pathological injury. In this study, the miRNAs and mRNAs expression profiles were comprehensively analyzed at 3rd, 5th and 7th days after infection with the NC group using RNA-seq, after the new SIV SW2783 was infected with the turbinate tissue of tree shrews. It was observed that miR-760-3p, miR-449b, miR-30e-3p, and miR-429 were significantly differentially expressed, and combined with functional analysis and interaction networks, it was speculated that these miRNAs might inhibit influenza virus replication in cells through signal transduction and transcriptional regulation. miR-324-3p, miR-1301, miR-103-1, miR-134-5p, miR-29a, miR-31, miR-16b, miR-34a, and miR-125b were low expression, and the analysis of their predicted target genes suggested that they were involved in the prevention of IAVs infection by regulating the expression of mRNAs related to immune signaling pathways. In addition, it is speculated that miR-106b-3p may be related to the cross-species infection potential of SW2783. The differential expression of the above miRNAs may affect viral replication and antiviral immunity in the cross-species transmission of SW2783 and the expression level changes with time of infection. Their abnormal expression may promote viral replication and immune escape, which is speculated to be of great significance in the cross-species transmission of SW2783. Although it remains to be determined whether these miRNAs play a role in regulating the immune response during IAVs infection, this study lays a foundation for the study of the molecular regulatory mechanism of miRNAs during IAVs infection and provides a valuable candidate biomarker for the intervention or control of influenza virus outbreaks in the population.

## Data Availability

The datasets generated and analyzed during the current study are available from the corresponding author upon reasonable request. Only the miRNA microarray data and mRNA sequencing data are not publicly available because these data contain unpublished information.

## Abbreviations

miRNA: MicroRNA
IAV: Influenza A virus
mRNA: Messenger RNA
DEMs: Differentially expressed miRNAs
DEGs: Differentially expressed mRNAs
SIV: Swine influenza virus
TRV: Triple recombinant virus
HPV: Human papilloma virus
HIV: Human immunodeficiency virus
dpi: Days post infection
GO: Gene ontology
KEGG: Kyoto encylopaedia of genes and genomes
HA: Haemagglutination
KO: KEGG orthology
RT-qPCR: Real-time quantitative PCR
hNEC: Human nasal epithelial cells
